# MEDICAL RESIDENCE IN ORTHOPEDICS AND TRAUMATOLOGY – NATIONAL OVERVIEW AND ANALYSIS OF EVALUATION CONCORDANCE BETWEEN CNRM/SBOT DURING THE COVID-19 PANDEMIC

**DOI:** 10.1590/1413-785220233102e260339

**Published:** 2023-06-09

**Authors:** VIVIANE CRISTINA ULIANA PETERLE, LUIZ KOITI KIMURA, PAULO EMILIANO BEZERRA, ANNE CAROLINE CASTRO PEREIRA, BRUNA PAIVA DE FRANÇA, NATHALIA MOURA RAMOS

**Affiliations:** 1. Universidade do Distrito Federal – UNIDF/Escola Superior de Ciências da Saúde (ESCS), Brasília, DF, Brazil. Secretária Executiva da Comissão Nacional de Residência Médica/CNRM/MEC.; Secretária Executiva da Comissão Nacional de Residência Médica; 2. Universidade de São Paulo, Orthopedics and Traumatology Institute of the Hospital das Clínicas (IOTHC), São Paulo, SP, Brazil. Presidente da Comissão Estadual de Residência Médica do Estado de São Paulo – CEREM/SP.; Comissão Estadual de Residência Médica do Estado de São Paulo; 3. Serviço de Ortopedia Hospital Regional do Paranoá (SES DF), Brasília, DF, Brazil.; 4. Centro Universitário de Brasília, Brasília, DF, Brazil.

**Keywords:** Medical Residency, Orthopedics, Medical Education, Health Systems, Residência Médica, Ortopedia, Educação Médica, Sistemas de Saúde

## Abstract

**Objective:**

Describe the national scenario of the orthopedics and traumatology Medical Residency Program (MRP) in 2020/2021, showing the distribution of vacancies by states and regions of Brazil, the number of residents and the percentage of agreement between the accredited services that offer the program by the Brazilian Society of Orthopedics and Traumatology (SBOT) and by the National Commission for Medical Residency linked to the Ministry of Education (CNRM/MEC).

**Methods:**

This is a descriptive, cross-sectional study. Data from the CNRM and SBOT system referring to residents attending orthopedics and traumatology Programs in 2020/2021 were analyzed.

**Results:**

In the analyzed period, there were 2.325 medical residents in orthopedics and traumatology in vacancies authorized by the CNRM/MEC in Brazil. The southeast region was predominant, with 57.2% of vacancies, totaling 1.331 residents. Compared to other regions, the south region with 16.9% (392), the northeast with 15.1% (351), the midwest with 7.7% (180), and the north with 3.1% (71). In addition, there was an accreditation agreement of 53.8% in evaluating services between the SBOT and CNRM, with distinctions among the states.

**Conclusion:**

The analysis showed differences between regions and states, considering the vacancies of PRM in orthopedics and traumatology and the concordance of evaluations by institutions accredited by MEC and SBOT. It is aim to work together with a view to qualifying and expanding residency programs for the training of specialist physicians, in accordance with the needs of the public health system and adequate medical practice, is necessary. The analysis during the pandemic period, associated with the restructuring of several health services, demonstrates the stability of the specialty in adverse scenarios. Level of evidence II; Economic And Decision Analyzes – Developing an Economic or Decision Model.

## RESUMO

**Objetivo:**

Descrever o cenário nacional do Programa de Residência Médica (PRM) em Ortopedia e Traumatologia em 2020/2021, período da maior incidência da covid-19, apontando a distribuição de vagas pelos Estados e Regiões do Brasil, o quantitativo de residentes em curso e a porcentagem de concordância entre os serviços credenciados pela Sociedade Brasileira de Ortopedia e Traumatologia (SBOT) e pela Comissão Nacional de Residência Médica vinculada ao Ministério da Educação (CNRM/MEC).

**Métodos:**

Estudo descritivo, transversal. Foram analisados dados do Sistema da CNRM e da SBOT referentes aos residentes cursando Ortopedia e Traumatologia durante o período declarado de pandemia no Brasil.

**Resultado:**

No período analisado haviam 2.325 médicos residentes cursando os PRM de Ortopedia e Traumatologia no Brasil em vagas autorizadas pela CNRM/MEC. Predomínio na região Sudeste, com 57,2% do total de vagas no Brasil, totalizando 1.331 residentes em comparação às outras regiões, com 16,9% (392) residentes na região Sul, 15,1% (351) no Nordeste, 7,7% (180) no Centro-Oeste e 3,1% (71) no Norte. Em relação à avaliação dos serviços realizada pela SBOT e pela CNRM, há uma concordância média de 53,8% entre o credenciamento por ambas, com também distinções entre as Unidades da Federação.

**Conclusão:**

A análise demonstrou diferenças entre regiões e estados em relação à oferta de vagas nos Programas de Residência em Ortopedia e Traumatologia, bem como quanto à concordância entre as avaliações das instituições credenciadas pela CNRM e/ou SBOT. Há necessidade de um trabalho conjunto entre ambas visando para ampliação e qualificação dos cenários de prática e preceptoria na formação do novo especialista, considerando as necessidades do SUS e o exercício da boa prática médica. A análise no período da pandemia, associado a reestruturação de muitos serviços de saúde, demostra a estabilidade da especialidade em cenários adversos. Nível de evidência II; Análises econômicas e de decisão – Desenvolvimento de modelo econômico ou de decisão.

## INTRODUCTION

Medical residency is a postgraduate education performed under in-service training, with a deepening in some medical areas. The National Medical Residency Commission (CNRM), linked to the Ministry of Education (MEC), is responsible for the organization, accreditation, and monitoring of the Medical Residency Programs (MRP) distributed throughout Brazil.^1^

Currently, these programs are developed in 55 medical specialties and 61 areas recognized by the Mixed Specialties Commission (CME), composed of the CNRM, Federal Council of Medicine (CFM), and the Brazilian Medical Association (AMB). orthopedics and traumatology MRP has three years duration and is a requirement to continue in Hand Surgery specialty.^2 - 4^

In the United States, the orthopedics residency is among the most competitive, without a simultaneous increase in vacancies.^5^ In Brazil, until 2018, also seemed to have an increased interest in the specialty, as shown in a nationwide study, recent graduates who intend to attend medical residency were asked their first option. Six specialties accounted for 53.3% of preferences, one of them being orthopedics and traumatology (5.2%).^6^ In parallel, another study showed data from 2010 to 2019 and a 96.9% increase in orthopedics vacancies in the country, from 487 to 959.^7^

However, there is still a need for studies that address the particularities of orthopedics and traumatology MRPs, such as the relationship between candidate and vacancy in the selection processes, weaknesses and qualities of the program, compliance with the competency matrices, among others.

The services offering the MRP are constantly evaluated following CNRM regulations.^8^ In 2018, the CNRM/MEC made resolutions that enable and encourage joint assessment between educational evaluators and specialty societies to increase qualification between the training services processes.

This measure eases the tension between the best possible understanding expected by specialty societies and CNRM/MEC regarding the importance and necessity of the evolution of the evaluation of Medical Residency Programs that unite several components, both of health services that are training scenarios for the specialist, and of the quality of care that impact good medical practice.

Therefore, the aim of this study is to describe the national scenario of orthopedics and traumatology MRPs in 2020/2021, showing the distribution of vacancies by states and regions of Brazil, the number of residents in total and per year, and the percentage of agreement between accredited services that offer the program by the Brazilian Society of Orthopedics and Traumatology (SBOT) and by the CNRM/MEC in 2020/2021. Also included in the data analysis was the MRP of hand surgery, a specialty that has the MRP of orthopedics and traumatology as a prerequisite.

## METHOD

This is a descriptive, cross-sectional study, conducted from a collection of pre-existing data, in electronic^9^ , based on the National Commission on Medical Residency System (SisCNRM) data through the MEC electronic portal (http://siscnrm.mec.gov.br/login/login), extracted between August 2020 and on April 2021. The database is generated by information provided by the Medical Residency Committees (COREME) in each institution responsible for the resident registration.

Variables with the number of residents attending orthopedics and traumatology specialty and hand surgery sub-specialty in 2020/2021 were selected to analyze the total, by state, of residents first-year (R1), residents second-year (R2), and residents third-year (R3) for the specialty and R1 and R2, for the sub-specialty. Institutions that listed the MRP status as “approved,” “overdue,” “diligence,” and “requirement” in SisCNRM were included.

As for the process of authorization with the Research Ethics Committee, the study is part of the research exempted from registration because it is a research that aims to deepen theoretical situations that emerge spontaneously in professional practice, which does not reveal data that can identify the individual.

The evaluation system of Medical Residency Programs for accreditation follows the regulations of the National Residency Commission regarding authorizing acts and is based on the pillar: structure-process-result, determined by on-site visit, document verification, interviews and analysis of the execution of the pedagogical project.

For comparison purposes, the services accredited by SBOT in 2020 were analyzed (https://sbot.org.br/wp-content/uploads/2020/08/Servicos_credenciados_2020.xls). The established criteria are determined by the Specialty Society and are based on their own criteria with main emphasis on the qualification of the teaching staff and scenarios for the implementation of the competence matrix.

In addition, a literature review was performed based on bibliographic research in the PUBMED database using the keywords “[MEDICAL RESIDENCY],” “[ORTHOPEDICS],” “[MEDICAL EDUCATION]” and their correspondents in Portuguese, and articles from the past five years were searched. A manual search was also performed on the SBOT website for discussions.

## RESULTS

In Brazil, there were 2.325 medical residents registered in the orthopedics and traumatology MRP in vacancies authorized by the CNRM/MEC in the analyzed period. The southeast region was predominant, totaling 1.331 residents, which represents 57.2% of the total number of residents in orthopedics and traumatology MRP in the country.

**Figure 1:**
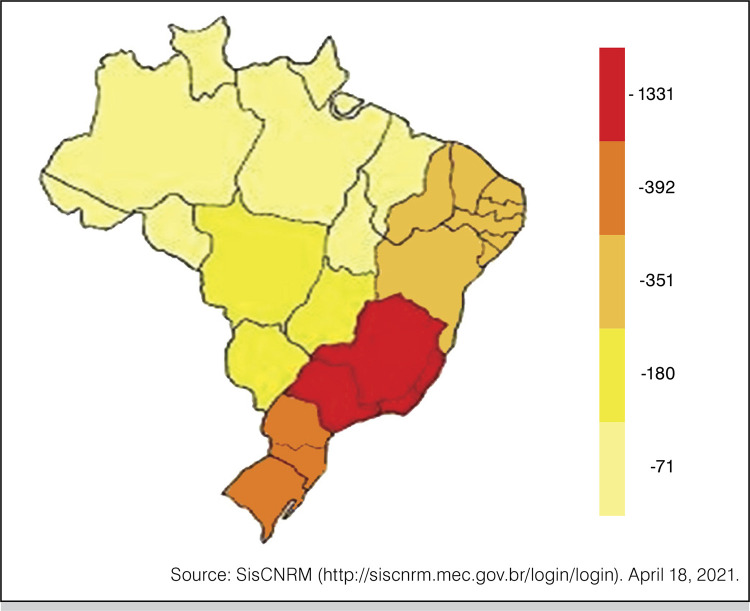
Number of resident physicians enrolled in Orthopedic and Traumatology Medical Residency Programs by region of Brazil in 2021.

Source: SisCNRM (http://siscnrm.mec.gov.br/login/login). April 18, 2021.

The discrepancy was notable compared to other regions, the south region with 16.9% (392), the northeast with 15.1% (351), the midwest with 7.7% (180), and the north with 3.1% (71). ( Figure 1 )

Of the total, 812 (34.9%) are enrolled in R1, 753 (32.4%) in R2, and 750 (32.3%) in R3, showing little waiver during the 3 years of the MRP. SisCNRM data also show 10 (0.4%) residents attending an additional fourth-year (R4) in various services in Brazil. ( Table 1 ), a low number given the possibility of extending training considering several specialized services in Brazil.

**Table 1:** Number of medical residents enrolled in each year of training in orthopedic and traumatology and hand surgery by state in 2021.

	Orthopedic and Traumatology					Hand Surgery		
State	R1	R2	R3	R4	Total	R1	R2	Total
AC	3	0	1	0	4	0	0	0
AL	10	5	7	0	22	0	0	0
AP	2	1	2	0	5	0	0	0
AM	8	6	7	0	21	0	0	0
BA	44	35	43	0	122	0	0	0
CE	15	15	14	0	44	0	0	0
DF	35	33	30	0	98	0	0	0
ES	9	9	10	0	28	1	0	1
GO	20	17	22	0	59	1	1	2
MA	7	6	6	0	19	0	0	0
MT	6	3	3	0	12	0	0	0
MS	4	3	4	0	11	0	0	0
MG	90	86	83	3	262	6	6	12
PA	8	8	8	0	24	1	2	3
PB	9	8	7	0	24	0	0	0
PR	70	62	56	0	188	3	3	6
PE	29	31	32	0	92	6	6	12
PI	6	5	4	0	15	0	0	0
RJ	71	71	68	1	211	7	8	15
RN	0	0	0	0	0	0	0	0
RS	46	42	38	0	126	3	3	6
RO	2	2	2	0	6	0	0	0
RR	1	3	2	0	6	0	0	0
SC	29	24	19	6	78	1	1	2
SE	6	4	3	0	13	0	0	0
SP	280	273	277	0	830	33	32	65
TO	2	1	2	0	6	0	0	0
Total	812	753	750	10	2325	62	62	124

AC: Acre; AL: Alagoas; AP: Amapá; AM: Amazonas; BA: Bahia; CE: Ceará; DF: Distrito Federal; ES: Espírito Santo; GO: Goiás; MA: Maranhão; MT: Mato Grosso; MS: Mato Grosso do Sul; MG: Minas Gerais; PA: Pará; PB: Paraíba; PR: Paraná; PE: Pernambuco; PI: Piauí; RJ: Rio de Janeiro; RN: Rio Grande do Norte; RS: Rio Grande do Sul; RO: Rondônia; RR: Roraima; SC: Santa Catarina; SP: São Paulo; SE: Sergipe; TO: Tocantins. Source: SisCNRM ( http://siscnrm.mec.gov.br/login/login ). April 18, 2021.

As for the distribution by state, São Paulo (SP) had the higher number of residents, with 830, corresponding to 35.7% of the total number of residents in the country, distributed between R1 280 (33.7%), R2 273 (32.9%) and R3 277 (33.4%). These data showed the residents concentration in this state, followed by the states of Minas Gerais (MG), with 262 (11.3%), and Rio de Janeiro (RJ), with 211 (9.1%). Along with SP, they justify the southeast region with the highest number of ongoing residents.

As for the states with the lowest numbers of residents, Tocantins (TO), Amapá (AP), and Acre (AC) accounted for 5, 5, and 4, respectively, followed by the Rio Grande do Norte (RN), as the only state that did not have residents attending the orthopedics MRP in the period.

Considering the distribution by Brazilian state between R1, R2, and R3, there was homogeneity between vacancies offered per year, without considerable discrepancies, following the verification of little dropout during the course regardless of the location.

Regarding the hand surgery MRP, 124 residents were registered. The highest number of vacancies remained in the southeast, with 75% (93), followed by the south 11.3% (14), northeast 9.7% (12), north 2.4% (3), and midwest 1.6% (2). ( Table 2 )

**Table 2:** Number of medical residents enrolled in orthopedic and traumatology and hand surgery in 2021.

	Orthopedic and Traumatology	Hand Surgery
**Region**	**Number of residents (%)**	**Number of residents (%)**
North	71 (3.1%)	3 (2.4%)
Northeast	351 (15.1%)	12 (9.7%)
Southeast	1.331 (57.2%)	93 (75%)
Midwest	180 (7.7%)	2 (1.6%)
South	392 (16.9%)	14 (11.3%)
Total	2,325	124

Source: SisCNRM (http://siscnrm.mec.gov.br/login/login). April 18, 2021.

SP state accounted for 65 (52%) residents in this sub-specialty, 33 R1, and 32 R2, followed by RJ, Pernambuco (PI), and Minas Gerais (MG) with 15, 12, and 12, respectively. Espírito Santo (ES), Goiás (GO), Pará (PB), Paraná (PR), Santa Catarina (SC) and Rio Grande do Sul (RS) had lower numbers, ranging from 1 to 8. The remaining states did not have residents attending hand surgery MRP. ( Table 1 )

As for the orthopedics and traumatology MRPs accredited by the CNRM/MEC in 2020, according to SisCNRM ( Table 3 ), Brazil had a total of 274 services, considering those in “approved,” “overdue,” “diligence,” and “exigency.”

**Table 3:** Number of orthopedic and traumatology Medical Residency Programs (CNRM/MEC) by state in 2020.

	Approved	Overdue	Deligence	Requirement	Total
aC	1	-	-	-	1
AL	3	-	1	1	5
AP	1	-	-	-	1
AP	1	-	-	1	2
BA	11	-	-	2	13
CE	5	-	-	2	7
DF	7	-	-	1	8
ES	2	-	-	2	4
GO	6	-	-	1	7
MA	4	-	-	-	4
MT	3	-	-	1	4
MS	1	-	-	-	1
MG	33	1	-	2	36
PA	3	-	-	-	3
PB	3	-	-	-	3
PR	22	-	-	4	26
PE	11	-	-	2	13
PI	1	-	-	1	2
RJ	27	-	1	4	32
RN	-	1	-	-	1
RS	14	1	1	1	17
RO	-	-	-	1	1
RR	-	-	-	1	1
SC	8	2	-	3	13
SE	3	-	-	-	3
SP	58	-	-	6	64
TO	2	-	-	-	2
TOTAL	230	5	3	36	274

AC: Acre; AL: Alagoas; AP: Amapá; AM: Amazonas; BA: Bahia; CE: Ceará; DF: Distrito Federal; ES: Espírito Santo; GO: Goiás; MA: Maranhão; MT: Mato Grosso; MS: Mato Grosso do Sul; MG: Minas Gerais; PA: Pará; PB: Paraíba; PR: Paraná; PE: Pernambuco; PI: Piauí; RJ: Rio de Janeiro; RN: Rio Grande do Norte; RS: Rio Grande do Sul; RO: Rondônia; RR: Roraima; SC: Santa Catarina; SP: São Paulo; SE: Sergipe; TO: Tocantins. Source: SisCNRM ( http://siscnrm.mec.gov.br/login/login ). October 31, 2020.

SP was the state with the highest number MRPs, with 64 (23.35%), followed by MG 36 (13.1%), RJ 32 (11.7%), PR 26 (9.5%), and RS 17 (6.2%), with the same sequence when analyzing the accredited vacancies by state, which reinforces the tendency and concentration in the southeast region, followed by the south, to the detriment of the others regions.

In the analyzed period, the North region presents the lowest concentration, totaling 11 accredited programs, with PA being the state with the highest number (3), followed by AM and TO (2) and AC, AP, Rondônia (RO), and Roraima (RR) (1). The number of programs in the other states varied between 4 and 13.

Regarding the different status of the MRPs within the CNRM, there were 230 accredited services as “approved”, 5 as “overdue,” 36 as “exigency,” and 3 as “diligence”. ( Table 3 )

Table 4 shows the number of services by states that offer training in orthopedics and traumatology accredited by the SBOT in 2020 and the quantity of MRP accredited by the CNRM/MEC, considering those in the abovementioned status.

**Table 4:** Number of accredited services by the National Commission on Medical Residency System (SisCNRM) compared to the Brazilian Society of Orthopedics and Traumatology (SBOT) offering orthopedics and traumatology Medical Residency Programs by state in 2020.

	Accredited services			
	SBOT	SISCNRM	BOTH	Total
AC	-	-	1	1
AL	-	4	1	5
AP	-	-	1	1
AM	-	-	2	2
BA	3	8	5	16
CE	1	2	4	7
DF	-	1	7	8
ES	-	-	4	4
GO	1	3	5	9
MA	1	2	2	5
MT	-	3	1	4
MS	-	-	1	1
MG	4	14	20	38
PA	2	3	-	5
PB	-	2	1	3
PR	3	12	14	29
PE	2	4	8	14
PI	1	1	1	3
RJ	1	15	17	33
RN	-	1	-	1
RS	2	7	9	18
RO	-	-	1	1
RR	-	1	-	1
SC	4	5	5	14
SE	-	2	1	3
SP	8	13	50	71
TO	-	2	-	2
TOTAL	34	104	161	299

AC: Acre; AL: Alagoas; AP: Amapá; AM: Amazonas; BA: Bahia; CE: Ceará; DF: Distrito Federal; ES: Espírito Santo; GO: Goiás; MA: Maranhão; MT: Mato Grosso; MS: Mato Grosso do Sul; MG: Minas Gerais; PA: Pará; PB: Paraíba; PR: Paraná; PE: Pernambuco; PI: Piauí; RJ: Rio de Janeiro; RN: Rio Grande do Norte; RS: Rio Grande do Sul; RO: Rondônia; RR: Roraima; SC: Santa Catarina; SP: São Paulo; SE: Sergipe; TO: Tocantins. Source: SisCNRM ( http://siscnrm.mec.gov.br/login/login ). October 31, 2020; Brazilian Society of Orthopedics and Traumatology (SBOT). October 31, 2020. ( https://sbot.org.br/wp-content/uploads/2020/08/Servicos_credenciados_2020.xls )

There are 299 accredited services for training in orthopedics in Brazil, with 161 institutions with assessments in common, representing on average 53.8% of agreement between the SBOT and MEC assessments for accreditation of services. However, it was also found that 34 (11.37%) institutions are accredited only by SBOT and 104 (34.78%) only by CNRM/ MEC.

Thirteen of the states (48.14%) had more than 50% agreement between both evaluations. 4 states in the North Region: AC, AP, AM, RO, in addition to the states of ES and MS, had 100% agreement. However, they also had a smaller number of institutions that offer residency programs.

Among the states with the highest number of institutions was the Distrito Federal (DF), which had the highest percentage of agreement (87.5%) among both accredited services by SBOT and CNRM/MEC. Then comes SP with 71 training services in total, with an agreement of 70.42%, with eight (11.3%) exclusively accredited by the SBOT, 13 (18.3%) by the CNRM/MEC, and 50 in both. Followed by Ceará (CE) and PE with 57.1%, GO with 55.5%, MG with 52.6%, and RJ with 51.5%.

Alagoas (AL) was in evidence among the states with less than 50% agreement, with five institutions offering training services, four exclusively by the CNRM and one by both, with 20% agreement. In addition, Mato Grosso (MT) (25%), Bahia (BA) (31.25%), PB, Piauí (PI) and Sergipe (SE) (33.33%), SC (35.7%), Maranhão (MA) (40%), PR (48.27 %) and RS (50%). Also noteworthy were the states that did not have joint accredited services, with a 0% agreement rate, such as PA, RN, RO, and TO.

The only states that had service accredited only by the CNRM/MEC were RN and RO with 1 and 2, respectively. The status of RO was “exigency.” The one in RN was “overdue,” which justifies the absence of residents in orthopedics. PA, in turn, had two institutions that were exclusive to the SBOT and three to the CNRM/MEC, not having a common record.

## DISCUSSION

The number of medical schools has been increasing in recent years in Brazil, but specialized, suitable, and structured a health service has not accompanied this number, making it impossible to increase the vacancies for residency programs.^10^

Although expanding these vacancies is necessary, it is essential to give attention to their distribution throughout the country. However, the expansion must be based on the conditionalities for its opening and accreditation, such as those required through MRPs periodic evaluations, to maintain the quality of training.^8^

The Medical Demography study^7^ showed in 2020 that the country counted a total of 17,906 physicians specializing in orthopedics and traumatology, with a ratio of 8.52 per 100,000 inhabitants. As for the distribution by region, there was a southeast predominance (52.9%), followed by the south (17%), northeast (16%), midwest (10%), and north (4.1%).

In addition to numbers, the hypotheses for the data presented must be discussed. Since only the orthopedics specialist can train the residents in this area, there is a clear relation between the concentration of specialists and the number of medical residency vacancies in the southeast region of Brazil. This can indicate quality in orthopedic physicians’ training, considering that preceptorship is essential for adequate training.

The southeast discrepant prevalence of 57.2% is too related to the concentration of more developed and traditional training centers in educational services – the first medical residency program registed in Brazil was in 1945 at the University of São Paulo – conditioned to suitable practice services for teaching, qualified training, technological resources for care and teaching, and budget for residency scholarship.^11^

In addition, it is questioned whether the socio-economic analysis influences the distribution of orthopedics and traumatology MRPs, considering that a large portion of residents leave their home state to specialize and do not return, when cursed in major urban centers.^12^ Only 38% of medical students and 23.7% of residents return home after completing their studies and/or specialization.^10 , 14^ The possible causes for this migration are the search for better working conditions and career opportunities.^10 , 12^ Thus, the concentration of MRPs also promotes the concentration of specialists in these regions.

Regarding the criteria for accreditation of medical residency programs, Decree No. 7,562 of 2011^8^ determines that the vacancies distribution, ideally following the epidemiological profile of the Unified Health System (SUS). As for this data, for example, data rates of hip fractures due to frailty in the elderly, Peterle et al^14^ found in their study that 28% of the records of femoral fractures in Brazil are the SP state. Therefore, there is compatibility between the health needs and training centers for orthopedics specialists in these regions.

However, what about other pathologies, such as orthopedic trauma, which is one of the main causes of care in emergency rooms. Or would it be the fact that patients also migrate to states and regions with greater structures for the treatment of their health problems? Should this epidemiological data be considered in the evaluation system of both institutions: CNRM and SBOT? How many specialists does Brazil need?

The CFM can only register as specialists (granting the Specialist Qualification Registration Certificate) physicians who present at least one of these two documents: Certificate of Completion of Medical Residency accredited by the National Commission for Medical Residency (CNRM) or Specialist Title granted by Brazilian Association or Society of the respective specialty.^15^

In orthopedics and traumatology, the resident must pass a demanding exam to receive the Specialist Title in Orthopedics and Traumatology (TEOT).^16 , 17^

CNRM/MEC Resolution No. 25^18^ from 2019 provides for cooperation between CNRM and medical specialty societies in the MRPs’ on-site evaluation visits, aiming to bring them closer together. However, as presented in this study, a greater relationship between the CNRM and SBOT is still necessary for better integration, since there is only 53.8% agreement in the accreditation between both assessments.

The study did not aim to evaluate the quality of MRP, interest of graduates of medical courses in the specialty, pedagogical project of MRP, idleness of vacancies or system of evaluation of the graduate is in MRP or training centers SBOT, but to signal, through quantitative data, assumptions that can unite and qualify the evaluation criteria between SBOT and CNRM, with a critical analysis, in addition to numerical discrepancy of data distribution of MRP and residents medical in the country.

## CONCLUSION

The analysis performed in this study showed significant differences between regions and states of Brazil considering the offer of vacancies in the orthopedics and traumatology MRPs and the distribution of institutions accredited by the MEC and/or SBOT, which follows the sociodemographic and the country’s health services. The services are concentrated in the southeast, followed by the south, compared to of the north region.

This may explain why the specialists stay in those regions where there is a greater concentration of orthopedics and traumatology services, both for the possibility of training in the specialty, and the chance to join the labor market in the area after finishing training. This also COULD explains why they do not return to their home state?

In turn, fulfilling the criteria which define medical residency under the responsibility of health institutions, university or not, and under the guidance of highly qualified ethical and professional physicians, the CNRM has been meeting its regulatory role based on pedagogical criteria regarding the accreditation of the most suitable services for the specialist training.

Thus, it is essential to identify the demographic arrangement of orthopedic training in the country, comparing it with the necessity of investment in specialized supplies and equipment and qualified professionals for the training of residents, as well as the epidemiological scenario of the incidence of pathologies to be treated by this specialty in the states and regions of Brazil.

In order to broaden the discussion, the debate on the training of orthopedic and traumatology specialists aims to contribute to the health systems management regarding more equitable investment planning in new services for the practice of the specialists, which will generate new job opportunities to encourage these specialists to establish their professional career in regions far from the larger centers.

## References

[1] Brasil Decreto nº 80.281, de 5 de setembro de 1977. Regulamenta a Residência Médica, cria a Comissão Nacional de Residência Médica e dá outras providências.

[2] Conselho Federal De Medicina Resolução Cfm Nº º 2330/2023 publicada em DOU de 15 de março de 2023. Diário Oficial da União.

[3] Brasil, Ministério da Educação Resolução Nº 22, De 8 De Abril De 2019. Aprova a matriz de competências dos Programas de Residência Médica em Ortopedia e Traumatologia. Diário Oficial Da União.

[4] Comissão Nacional De Residência Médica Resolução Cnrm Nº 05/2002.

[5] Nasreddine AY, Gallo R (2019). Applying to Orthopaedic Residency and Matching Rates: Analysis and Review of the Past 25 Years. J Bone Joint Surg Am.

[6] Scheffer M, Cassenote A, Guilloux AGA, Miotto BA, Mainardi GM (2018). Demografia Médica no Brasil 2018.

[7] Scheffer M, Cassenote A, Guerra A, Guilloux AGA, Brandão APD, Miotto BA (2020). Demografia Médica no Brasil 2020.

[8] Brasil Decreto Nº 7.562, de 15 de setembro de 2011. Dispõe sobre a Comissão Nacional de Residência Médica e o exercício das funções de regulação, supervisão e avaliação de instituições que ofertam residência médica e de programas de residência médica.

[9] Ministério da Saúde, Conselho Nacional de Saúde Resolução Nº 674, publicada em DOU de 6 de maio de 2022. Diário Oficial da União.

[10] Chaves HL, Borges LB, Guimarães DC, Cavalcanti LPG (2013). Vagas para residência médica no Brasil: Onde estão e o que é avaliado. Rev Bras Educ Med.

[11] Conselho Nacional Dos Secretários De Saúde Recorte Demográfico da Residência Médica Brasileira em 2019. Revista Consensus on-line.

[12] Logullo P (2016). O mapa das residências médicas em ortopedia no país. Jornal da SBOT.

[13] Scheffer M (2013). Demografia Médica no Brasil: cenários e indicadores de distribuição.

[14] Peterle VCU, Geber JC, Darwin W, Lima AV, Bezerra PE, Novaes MRCG (2020). Indicadores De Morbidade E Mortalidade Por Fraturas De Fêmur Em Idosos: Análise De Uma Década Em Hospitais Brasileiros. Acta Ortop Bras.

[15] Conselho Federal de Medicina Resolução CFM nº. 1785/2006. D.O.U. 22 de junho de 2006, Seção I.

[16] Jornal da SBOT Nº 109 Jan/Fev/Março 2013.

[17] Brasil Decreto Nº 8.516, de 10 de setembro de 2015. Regulamenta a formação do Cadastro Nacional de Especialistas de que tratam o § 4º e § 5º do art. 1º da Lei nº 6.932, de 7 de julho de 1981, e o art. 35 da Lei nº 12.871, de 22 de outubro de 2013.

[18] Brasil, Ministério da Educação Resolução Nº 25, de 16 de abril de 2019. Dispõe sobre a cooperação entre a CNRM e as sociedades médicas de especialidades nas visitas de avaliação in loco dos Programas de Residência Médica no Brasil.

